# Transcriptome analysis of differentially expressed genes involved in selenium accumulation in tea plant (*Camellia sinensis*)

**DOI:** 10.1371/journal.pone.0197506

**Published:** 2018-06-01

**Authors:** Dan Cao, Yanli Liu, Linlong Ma, Xiaofang Jin, Guiyi Guo, Rongrong Tan, Zheng Liu, Lin Zheng, Fei Ye, Wei Liu

**Affiliations:** 1 Fruit and Tea Research Institute, Hubei Academy of Agricultural Sciences, Wuhan, Hubei, China; 2 Henan Key Laboratory of Tea Comprehensive utilization in South Henan, Xinyang Agriculture and Forestry University, Xinyang, Henan, China; Youngstown State University, UNITED STATES

## Abstract

Tea plant (*Camellia sinensis*) has strong enrichment ability for selenium (Se). Selenite is the main form of Se absorbed and utilized by tea plant. However, the mechanism of selenite absorption and accumulation in tea plant is still unknown. In this study, RNA sequencing (RNA-seq) was used to perform transcriptomic analysis on the molecular mechanism of selenite absorption and accumulation in tea plant. 397.98 million high-quality reads were obtained and assembled into 168,212 unigenes, 89,605 of which were extensively annotated. There were 60,582 and 1,362 differentially expressed genes (DEGs) in roots and leaves, respectively. RNA-seq results were further validated by quantitative RT-PCR. Based on GO terms, the unigenes were mainly involved in cell, binding and metabolic process. KEGG pathway enrichment analysis showed that predominant pathways included ribosome and protein processing in endoplasmic reticulum. Further analysis revealed that sulfur metabolism, glutathione metabolism, selenocompound metabolism and plant hormone signal transduction responded to selenite in tea plant. Additionally, a large number of genes of higher expressions associated with phosphate transporters, sulfur assimilation, antioxidant enzymes, antioxidant substances and responses to ethylene and jasmonic acid were identified. Stress-related plant hormones might play a signaling role in promoting sulfate/selenite uptake and assimilation in tea plant. Moreover, some other Se accumulation mechanisms of tea plant were found. Our study provides a possibility for controlling Se accumulation in tea plant through bio-technologies and will be helpful for breeding new tea cultivars.

## Introduction

Selenium (Se) is an essential micronutrient for human and animals. However, there is a very narrow concentration margin between harmful and beneficial doses of Se [1]. Excessive Se can cause poisoning [2], while Se deficiency may result in some endemic diseases such as Kashin-Beck disease and Keshan disease [3]. Plants can remove Se from natural or polluted high-Se areas and can improve Se nutrition for human as food sources [4]. On the past years several biofortification experiments on vegetables have been carried out and a large body of evidence concerning the effects of Se supplementation has been collected [5–10]. Tea, one of the most popular non-alcoholic beverages in the world and a cash crop widely cultivated not only in Se-rich areas such as Enshi, Hubei province and Ziyang, Shanxi province but also in Se-deficient areas, contains many medicinal ingredients like tea polyphenols, caffeine and amino acids and can reduce fat, lose weight, lower blood sugar, improve immunity and so on [11–13]. Meanwhile, tea is superior to some plant foods in terms of Se content and can provide effective organic Se for human body [14–17]. However, the mechanisms of Se absorption, transport and metabolism in tea plant are still not clear, and relevant studies will be helpful for Se biofortification and improvement of human nutrition.

Selenate (VI) and selenite (IV) are predominant forms of Se in plants. The forms of Se in soil are greatly affected by redox potential and pH. A study of thermodynamic calculations showed that selenate was the main existence form at the high redox state (pE+pH>15.0), and selenite mainly existed in the medium redox range (7.5<pE+pH<15.0) [18]. The mechanism of selenate uptake in plants is well understood. Se and sulfur (S) are similar chemically, and selenate was absorbed by sulfate transporters [19–20]. For example, *AtSultrl1;2* encoding one of the high-affinity sulphate transporter genes was assayed via isolating *Arabidopsis thaliana* mutants [21]. Several studies have also clarified that *Sultr2*, a low-affinity sulphate transporter gene, was involved in translocation from roots to shoots, irrespective of Se supply or sulfate starvation [22–23]. Unlike selenate, the mechanism of selenite absorption by plants is still unclear. It was found that the absorption of selenite was unaffected by sulphate [24–25]. The genes may be indispensable for both selenate and selenite absorption by roots. Early studies suggested that selenite was assimilated by passive diffusion into roots [26]. Later, another research found that a silicon(Si) influx transporter, *OsNIP2;1* (a nodulin 26-like intrinsic membrane protein subfamily of aquaporins), was related to selenite uptake [27]. However, two phosphate transporters, *OsPT2* and *OsPT8*, were also verified to be associated with the absorption of selenite [28–29]. Till now, there is no unified statement. A gene, *CsSUL3*.*5*, was cloned from the roots of tea plant. The bioinformatics analysis showed that it belonged to the sulfate transporter family and selenate treatment could induce its expression [30]. In spite of some advances in physiology studies of selenite accumulation in tea plant [31–32], the genes related with selenite uptake, transport and assimilation and the whole gene network are still not well known and expect for further researches.

With the rapid development of next-generation sequencing technologies, RNA-Seq has quickly become a powerful approach for studying transcriptional regulation systematically. In this study, RNA-seq technology was used to identify genes involved in selenite uptake and metabolism in tea plant. Transcriptome analysis was performed on both tender roots and young leaf tissues of tea plant with or without selenite treatment. Our study is not only useful to analyze the molecular mechanism of tea plant in response to selenite but will also be helpful for breeding new tea cultivars.

## Materials and methods

### Experimental materials and culturing conditions

The one-year-old cuttings (Echa 1) were provided by tea germplasm resource nursery in Hubei province. Uniform seedlings were pre-cultured until new roots grew. In early studies, Se concentrations in nutrient solutions varied from 0, 0.015, 0.025, 0.050, 0.100, 0.200 to 0.400 mM. With the enhancement of Se concentration in nutrient solutions, the content of Se in roots increased gradually. The absorption and accumulation of Se was fast in the low Se concentration range (0.015–0.10 mM). Se could still be taken up in high-Se environment, but at a low rate. However, the absorption rate of Se increased again at very high Se concentrations. This might be because cell membranes were destroyed and Se entered cells directly. However, in spite of this, there were no toxicity symptoms on the leaves (Fig B in S1 Fig). When the concentration of Se was 0.05 mM, tea plants grew best, and the content of Se in roots increased at a very fast rate (Fig A in S1 Fig), which was similar to other studies [32, 33]. Thus, Se was supplied as selenite at this concentration in this study, with zero selenite concentration as the control. Three replicates were performed with 20 seedlings per pot containing 10 L nutrient solution. The nutrient solution was made based on a modified Hoagland’s formula [34], that is, 1/3 dilution of a full solution containing 5.00 mM Ca(NO_3_)_2_·4H_2_O, 5.00 mM KNO_3_, 1.00 mM NH_4_NO_3_, 1.00 mM KH_2_PO_4_, 2.00 mM MgSO_4_·7H_2_O, 0.10 mM FeSO_4_·7H_2_O, 0.10 mM C_10_H_14_N_2_Na_2_O_8_·2H_2_0, 0.01 mM KI, 0.10 mM H_3_BO_3_, 0.15 mM MnSO_4_, 0.03 mM ZnSO_4_·7H_2_O, 1.00 μM Na_2_MoO_4_·2H_2_O, 0.10 μM CuSO_4_·5H_2_O, 0.20 μM CoCl_2_, with or without 0.05 mM selenite. The pH value was adjusted to 5.0 every day with 1mM HCl or 1mM NaOH. The nutrient solution was aerated continuously and renewed once a week. The experiment was conducted in a greenhouse with a 300–350μmol m-2s-1photon flux density for 12 h/d, and at day/night temperature of 30/24°C and relative humidity of 80%. A month later, the bud spread into five new leaves. Seedlings were harvested separately, and the roots were rinsed with the desorption solution. Leaf tissues comprised two leaves and a bud, and tender roots consisted of the latest and the second lateral roots. In the end, all samples were frozen in liquid nitrogen and stored at -80°C.

### Total Se analysis

The roots and leaves were digested for 12 h with concentrated HNO_3_-HClO_4_ (4:1,v/v) at first, and then completely digested at 180°C until the digestion solution became colourless. After cooling, 6 M HCl was added to reduce Se^6+^ to Se^4+^. Subsequently, the digested samples were diluted and transferred into a 25 mL volumetric flask with double deionized water. Se concentration was determined with an atomic fluorescence apectrometry (AF-610B, Beijing Ruili Instrument Co., Ltd., China). The standard reference materials and a blank were digested for quality control.

### Determination of hydrogen peroxide (H_2_O_2_) content and antioxidant enzymes’ activities

In order to determine the content of hydrogen peroxide, 0.5 g roots of the control and treated seedlings were homogenized in an ice bath with 5 mL 0.1% (w/v) trichloroacetic acid. The homogenate was centrifuged at 12000 g for 15 min at 4°C and 0.5 mL of the supernatant was added to 1 mL of 1 M KI and 0.5 mL of 10 mM potassium phosphate buffer (pH 7.0). The absorbance was measured at 390 nm [35].

For the determination of the activities of antioxidant enzymes, 0.5 g roots were homogenized in an ice bath with 4.0 mL of the extraction solution comprising 0.05 M phosphate buffer (pH 7.0), 1.0% (w/v) polyvinypyrrolidone, 1 mM ethyleneglycol-bis(beta-aminoethylether)-N,N'-tetraacetic acid, 1 mM phenylmethylsulfonyl fluoride, 1 mM dithiothreitol and 0.2% Triton X-100 (v/v). Insoluble materials were removed by centrifugation at 12000 g for 20 min at 4°C. The supernatant was kept at -20°C for further measurement. For the assay of ascorbate peroxidase (APX), 50 μL of the enzyme extract was added to 2.9 mL of the reaction buffer containing 50 mM Tris-HCl (pH 7.0), 0.1 mM ethylenediaminetetraacetic acid and 0.1 mM H_2_O_2_, and then 50 μL of 30 mM ascorbic acid was added. The absorbance of the mixture was recorded at 290 nm after 10–60 s [36]. For the estimation of glutathione peroxidase (GPX), 50 μL of the enzyme extract was added to 2.9 mL of the mixture containing 50 mM Tris-HCl (pH 7.0), 0.1 mM ethylenediaminetetraacetic acid, 10 mM guaiacol and 5 mM H_2_O_2_. The absorbance of the mixture was measured at 470 nm after 0.5–3.5 min [37]. For the determination of catalase (CAT), 50 μL of the enzyme extract was added to 2.9 mL of the mixture comprising 50 mM Tris-HCl (pH 7.0) and 0.1 mM ethylenediaminetetraacetic acid, and then 50 μL of 750 mM H_2_O_2_ was added. The absorbance of the mixture was measured at 240 nm after 0.5–3.5 min [38].

### RNA isolation, library construction and sequencing for RNA-Seq

The total RNA was extracted from roots and leaf tissues separately using OminiPlant RNA Kit (ComWin Biotech, Beijing, china). The RNA integrity was verified by RNase-free agarose gel electrophoresis, the RNA purity was quantified by Nanodrop 2000 (Thermo, USA), the RNA concentration was measured using a 2100 Bioanalyzer (Agilent Technologies, USA), and each treatment was done in triplicate biologically. All twelve samples had a RNA integrity number (RIN) >7.5, and a RNA concentration ≥100 ng/μL.

The total RNA was extracted and qualified, and an equal amount of RNA (3μg) was used to construct the sequencing library with a NEBNext® Ultra™ Directional RNA Library Prep Kit for Illumina (NEB, USA) according to the manufacturer’s recommendations. In brief, mRNA was enriched by Oligo (dT) beads from total RNA at first, and then fragmented into short ones through fragmentation buffer and reversely transcripted into cDNA with random primers. Using DNA polymerase I, dNTP, RNase H and buffer, the second-strand cDNA was synthesized. The cDNA fragments were purified with QiaQuick PCR extraction kit, end repaired, added, and ligated to illumina sequencing adapters. Finally, the products were selected by agarose gel electrophoresis, PCR amplified, and paird-end sequenced with an Illumina HiSeqTM2000 system of Gene Denovo Biotechnology Co., Ltd. (Guangzhou, China).

### Sequencing analysis, *de novo* transcripts assembly and functional annotation

After sequencing, raw reads were filtered according to three rules: (1) removing reads containing adapters, (2) removing reads containing more than 10% of unknown uncleotides, (3) removing low-quality reads containing more than 50% of low-quality (Q-value≤10) bases. The high-quality clean reads were mapped to ribosome RNA (rRNA) to identify residual rRNA reads. The rRNA removed reads were *de novo* assembled by short reads assembling program-Trinity (v2.1.1) [39], and statistics for length distribution of assembled unigenes were made. The unigene expression was calculated and normalized to RPKM (reads per kb per million reads) [40] based on RPKM (A) = (1000000*C)/(N*L/1000) (where C, N stand for the number of reads uniquely mapped to unigene A and all unigenes respectively, and L stands for the length of unigene A). This approach could eliminate the influences of unigene length and sequencing discrepancies on the calculation of unigene expression. The false discovery rate (FDR) was calculated to adjust the threshold of p value [41]. The unigenes with FDR<0.05 and |log2FC|>1 were considered DEGs.

To annotate the unigenes, Blastx program (v2.2.29+) with an E-value threshold of 1e-5 was used to find the unigenes against NCBI non redundant protein (Nr) database, the Swiss-prot protein database, the kyoto encyclopedia of genes and genomes (KEGG) database, and the clusters of orthologous groups (COG) database. According to the best alignment results, protein functional annotations could be obtained. Based on the results of Nr annotation, Gene Ontology (GO) annotation of the unigenes was analyzed by blast2go software [42], and functional classification of the unigenes was performed using WEGO program [43].

### Protein coding region prediction and transcription factor analysis

The unigenes coding sequence (CDS) was predicted by Blastx and ESTscan. At first, the unigenes were aligned by Blastx (E-value cutoff 10–5) to protein databases in an order of priority from NR, Swiss-prot, KEGG to COG/KOG. Then, the best alignment results were chosen to decide the sequence direction of the unigenes. If a unigene could not match with any protein database, protein coding sequence and sequence direction would be confirmed using ESTScan [44] program. Moreover, HMMER (v3.0) was used to predict transcription factor (TF) based on the plant TF databases [45].

### Real-time quantitative PCR assay

In order to confirm the reliability of RNA-seq results, 15 representative responsive genes were chosen for qRT-PCR using KAPA SYBR® FAST qPCR Master Mix (KAPA Biosystems, Woburn, MA., USA) with an ABI 7500 Real-Time PCR system according to the manufacture’s instructions. β-actin and dehyde-3-phosphate dehydrogenase (GAPDH) were used as reference genes. To derive the relative expression value, the delta-delta CT method [46] was adopted. The sequences of the primers were listed in S1 Table.

## Results

### Se content

**Fig 1:**
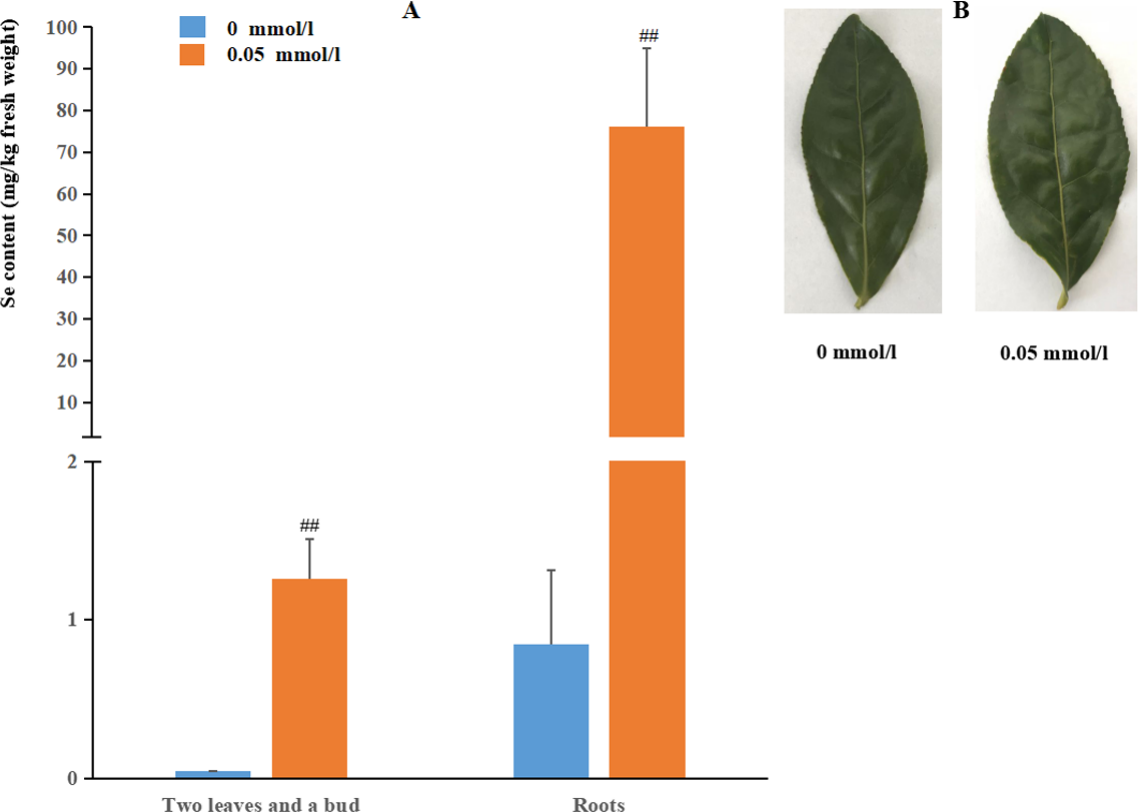
Changes of tea plants in response to selenite treatment. (A) Se contents in tea plant roots and leaves between control and treatment groups. ## represents significant difference at 0.01 level. (B) Morphological appearance of tea plant leaves treated with or without selenite.

Se concentration analysis implied that Se contents in both roots and shoots showed increased significantly with selenite treatment (p<0.01). Moreover, Se content in roots was much higher than that in shoots, which was consistent with the findings in other species (Fig 1) [10, 25, 47–48]. This is probably because selenite can be easily transformed into the organic form in roots once absorbed by plants [49].

### H_2_O_2_ content and three antioxidant enzyme activities

H_2_O_2_, a signaling molecule, plays an important role in oxidative response [50], but excessive H_2_O_2_ can disrupt the antioxidant enzyme system. APX, CAT and many other compounds of plant cells are important to maintain an appropriate concentration of H_2_O_2_ at different developmental stages [51]. Table 1 showed the behaviors of H_2_O_2_, APX, GPX and CAT in the roots of tea plants exposed to two treatments. Selenite treatment resulted in a reduction of H_2_O_2_ content. The activities of APX and GPX were improved, whereas the activity of CAT remained almost unchanged with selenite treatment. It has been reported that APX and GPX could reduce H_2_O_2_ content [52–53], which could explain the lower level of H_2_O_2_ accumulation. Consequently, the higher activities of APX and GPX in the roots in the present study suggested their importance of improving Se tolerance.

**Table 1:** Effect of selenite treatment on the content of H_2_O_2_ and three antioxidant enzyme activities in the roots of tea plants.

Selenite concentration (mM)	H_2_O_2_ (μg/gFW)	APX(μmol ASA/min/g FW)	GPX(μmol guaiacol/min/ FW)	CAT(μmol H_2_O_2_/min/g FW)
0	44.953±5.89	1.998±0.25	9.626±2.53	10.333±0.00
0.05	18.420±3.52^##^	2.826±0.31^#^	12.573±3.66^##^	11.167±0.00

# represents significant difference at 0.05 level, and

## represents significant difference at 0.01 level

### RNA-seq and *de nove* assembly

RNA-Seq of 12 libraries from young leaf tissues and tender roots with and without selenite treatment (three replicates respectively) resulted in 411.22 million reads with more than 95% exhibiting a quality score of Q20 (S2 Table). Using Trinity, 397.98 million high-quality clean reads were *de novo* assembled into 168,212 unigenes (S3 Table) ranging from 201 to 17,995 bp, with a mean length of 677 bp and a N50 length of 997 bp. All unigenes were longer than 200 bp and 54.43% (6,502) of unigenes were longer than 1,000 bp (S2 Fig), which indicated a high-quality sequence, and could be used for further analysis.

### Gene functional annotation and classification

**Fig 2:**
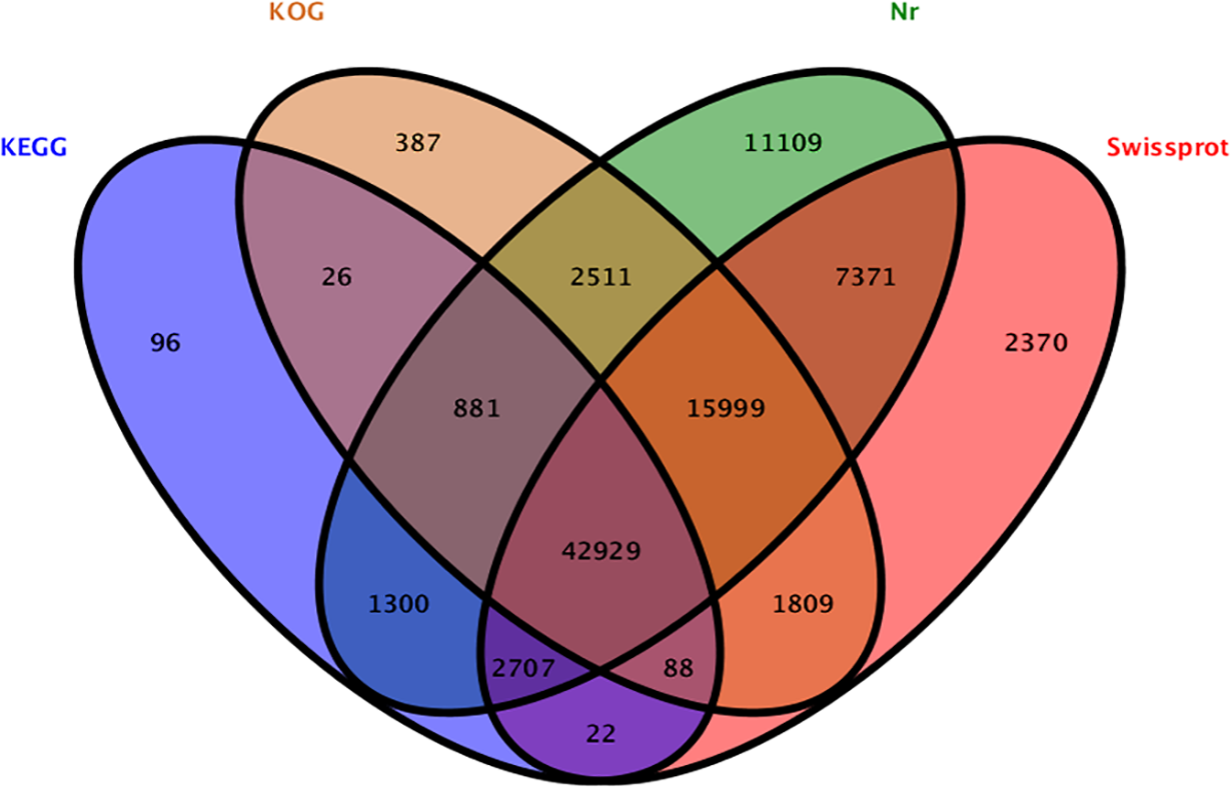
Venn diagram showing BLAST results of tea plants transcriptome against four databases. The assembled unigenes were used in BLAST searches against four pubic databases. The number of unigenes with significant hits against four database is shown at each intersection of Venn diagram.

A total of 89,605 unigenes were annotated in the four protein databases, among which 84,807 unigenes were annotated uniquely in NR database, 73,295 unigenes in Swiss-prot database, 64,630 unigenes in COG/KOG database, 48,049 unigenes in KEGG database, respectively. In 168,212 high-quality unigenes, 42,929 (25.52%) unigenes matched with all four databases and 84,807 (50.42%) unigenes matched with at least one database (Fig 2).

The maximum number of BLASTX top hits for the best group representatives was found with *Vitis vinifera* (8.98%), followed by *Theobroma cacao* (3.00%), *Klebsormidium flaccidum* (2.75%), *Chrysochromulina sp*. *CCMP291* (2.69%), *Nannochloropsis gaditana* (2.61%), and *Galdieria sulphuraria* (2.33%), suggesting that the genome of *Camellia sinensis* was more closely related to *Vitis vinifera* than any other plant genomes.

**Fig 3:**
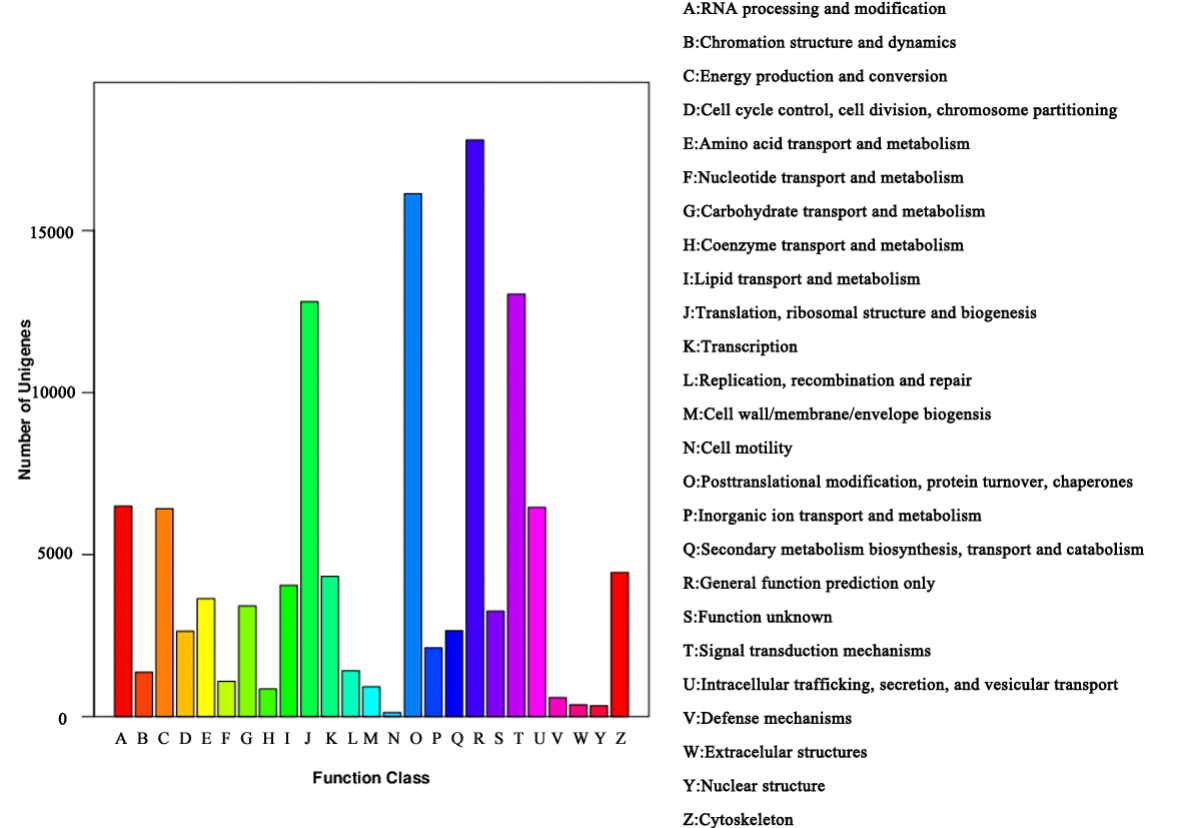
KOG functional classification of tea plants transcriptome. In total, 64,630 unigenes were classified into 25 KOG clusters. y-axis represents the number of unigenes, and the capital letters on x-axis represent KOG categories as listed at right of histogram.

In the unigene set of tea plants, 64,630 (38.42%) unigenes were categorized into 25 KOG clusters (Fig 3), and the five largest ones were: (1) general function prediction only, (2) post-translational modification, protein turnover, and chaperones, (3) signal transduction mechanisms, (4) translation, ribosomal structure and biogenesis, (5) RNA processing and modification.

### Protein coding sequence prediction and transcription factors analysis

Using the BLASTx protein database, a total of 88,624 unigenes CDSs were tested, among which 6,054 (6.83%) unigenes were longer than 500 bp, and 795 unigenes were longer than 1,000 bp. Another 15,104 unigenes CDSs could not match with the above-mentioned database. In total, 2,244 TF genes were identified as Se vs the control, which were classified into 57 families. In terms of the number of genes, top 10 were C2H2, bZIP, bHLH, C3H, MYB_related, ERF, MYB, NAC, GRAS, WRKY.

### Differentially expressed genes in response to Se

After selenite treatment, 21,588/38,994 genes were up/down-regulated in roots, 231/1,131 DEGs were up/down-regulated in leaves (P<0.05, false discovery rate [FDR]<0.05, |log2FC|>1) (S3 Fig). Among the significantly expressed genes in roots, only 423 genes were also induced in leaves, of which 174/249 genes were up-and down-regulated. The difference of DEGs indicated that the genes in response to selenite were different in roots and leaves. More genes were found in roots than in leaves, which reflected the stimuli imposed by selenite as it was transported in root cells. After all, the roots were where selenite was first perceived by plants.

### GO enrichment analysis of differentially expressed genes

**Fig 4:**
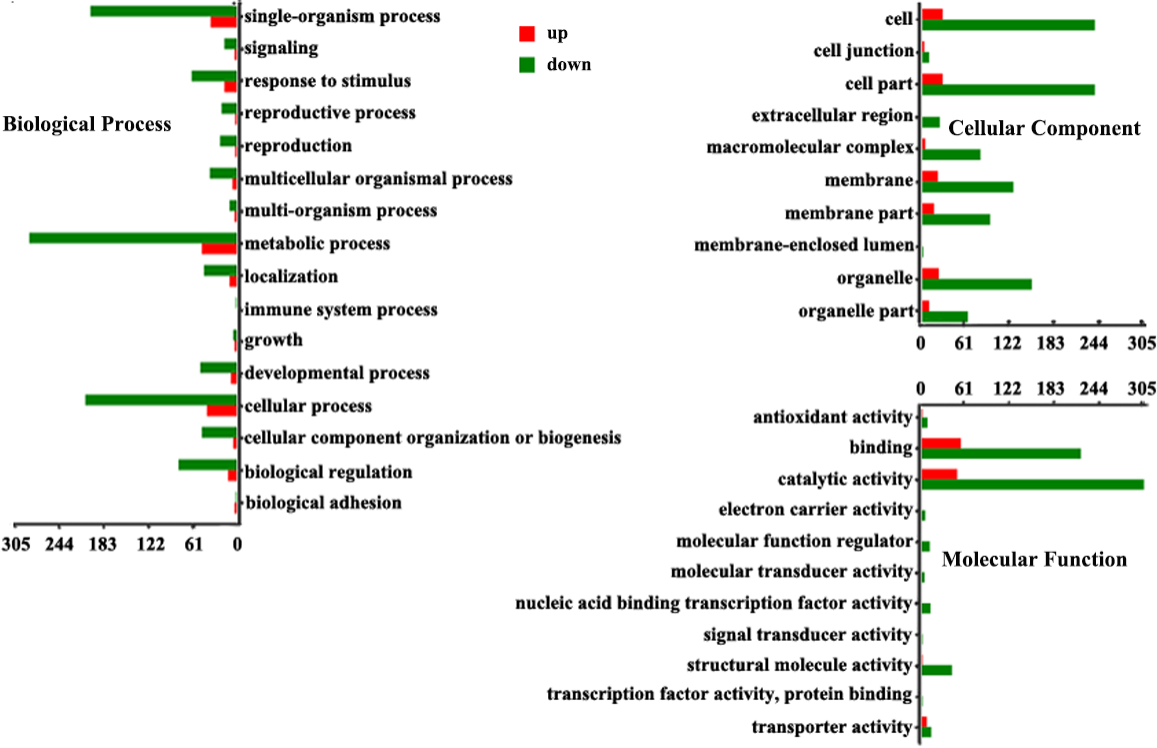
Function classifications of GO terms of tea plant roots transcripts. The x-axis indicates the number of genes in a subcategory, and the y-axis indicates the different subcategories.

Based on GO terms, a total of 53,131 unigenes could be classified into 3 gene ontology (GO) categories, i.e., biological process, molecular function and cellular component. There were more unigenes classified into cellular component than the other two categories. GO enrichment analysis of DEGs found that the most enriched ones were metabolic process, catalytic activity, and cell in both roots and leaves (Fig 4 and S4 Fig)

### KEGG pathway enrichment analysis of DEGs

**Fig 5:**
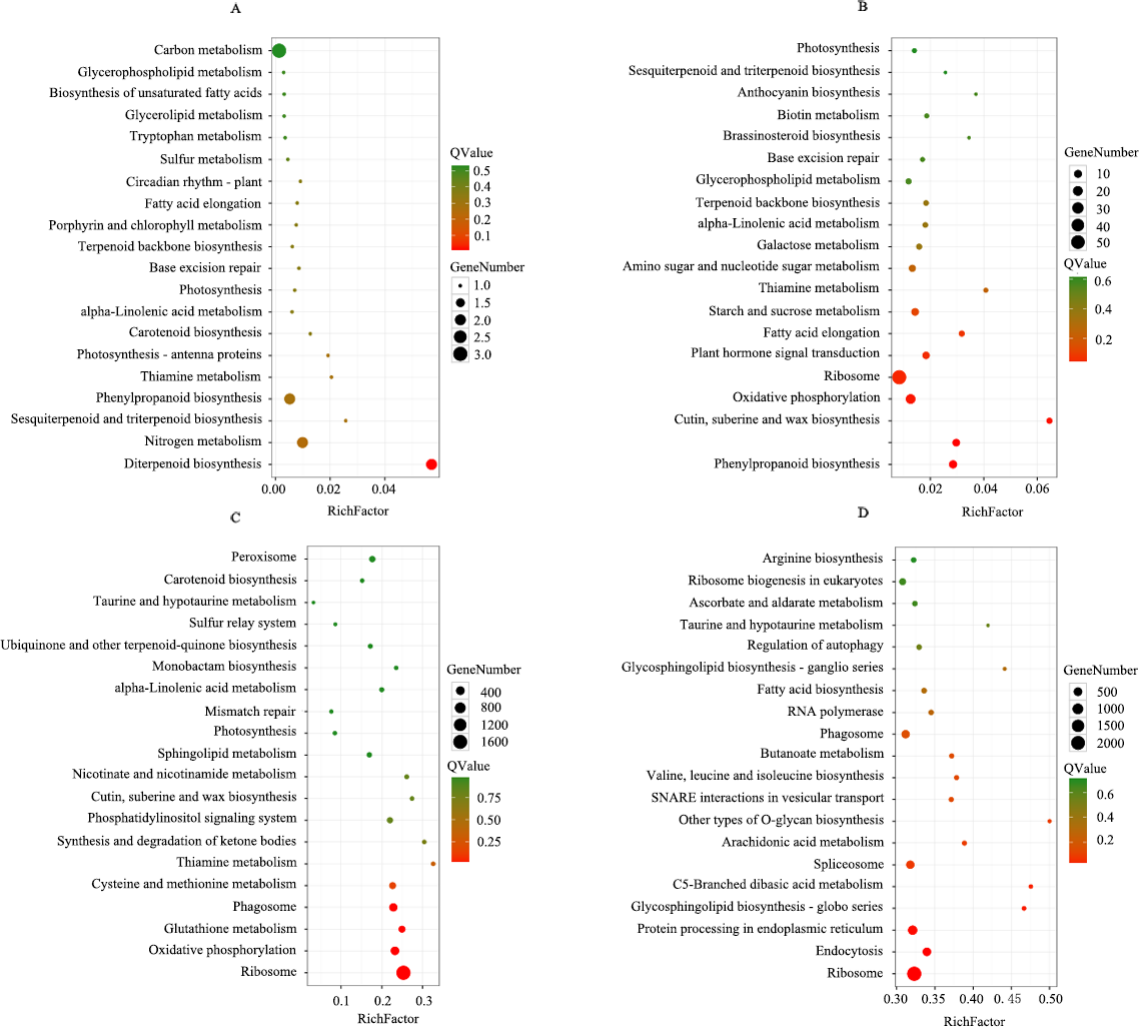
KEGG enrichment analysis of DEGs in tea plants. **(**A) Up-regulated genes in the leaves, (B) down-regulated genes in the leaves, (C) Up-regulated genes in the roots, (D)down-regulated genes in the roots. The x-axis indicates the enrichment factor, and the y-axis shows the KEGG pathway. The color of dot represents the Q-value, and the size of the dot represents the number of genes.

KEGG pathway enrichment analysis was performed on the up- and down-regulated genes. In leaves, the up- and down-regulated genes were enriched on 39 and 67 pathways, respectively. The pathways where the up-regulated genes were mostly enriched were carbon metabolism (3 unigenes), nitrogen metabolism (2 unigenes) and phenylpropanoid biosynthesis (2 unigenes) (Fig 5A), and the pathways where the down-regulated genes were mostly enriched were ribosome (55 unigenes), oxidative phosphorylation (20 unigenes) and phenylpropanoid biosynthesis (11 unigenes) (Fig 5B). In roots, the up- and down-regulated genes were enriched on 126 and 127 metabolic pathways, respectively. The pathways for significant enrichment of the up-regulated genes were ribosome (1664 unigenes), oxidative phosphorylation (366 unigenes) and phagosome (327 unigenes) (Fig 5C), while the pathways for significant enrichment of the down-regulated genes were ribosome (2121 unigenes), protein processing in endoplasmic reticulum (664 unigenes) and endocytosis (463 unigenes) (Fig 5D). DEGs in both roots and leaves were mainly enriched in ribosome (52 unigenes) and oxidative phosphorylation (14 unigenes). It is noteworthy that 46, 164, 40, 169, 63, 112 of the up-regulated genes in roots were respectively enriched in sulfur metabolism, cysteine and methionine metabolism, selenocompound metabolism, glutathione metabolism, plant hormone signal transduction, plant-pathogen interaction, indicating that these pathways were activated by selenite (S4 Table).

### RT-PCR analysis

**Fig 6:**
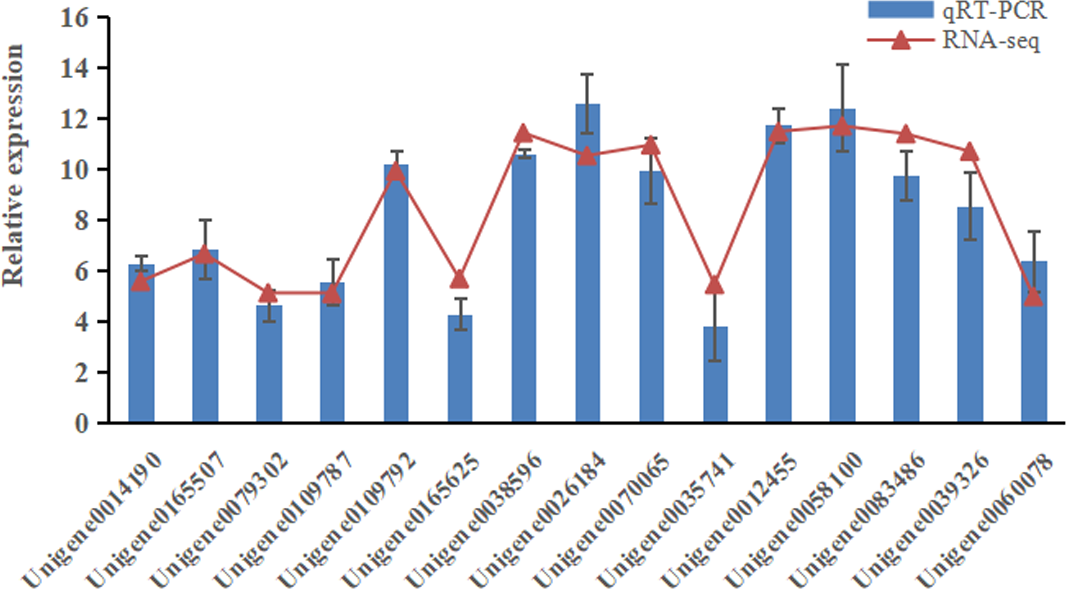
Verification of relative expression levels of DEGs by qRT-PCR. Red lines represent the RNA-seq results, while blue bars represent the qRT-PCR results. All qRT-PCR analyses were done in three biological replicates and four technical replicates. Error bars represent standard deviation.

To confirm RNA-seq data, 15 genes related with uptake and metabolism, defense and transcription factors in the roots were selected for qRT-PCR analysis. The expression tendency of these genes agreed well with RNA-seq results, which suggested that RNA-seq results were pretty reliable (Fig 6).

## Discussion

Tea plant can enrich Se from the external environment, and the bioavailable form in the leaves is the water-soluble Se. In our study, the content of two leaves and a bud was about 4.67 μg/g (dry weight), based on that water was 73% in the fresh leaves. The dietary reference intake of Se is 50 μg/d for adult humans in China, according to Chinese Nutrition Society. Normally, a healthy adult may drink 6~10g tea per day, and the leaching rate is up to 46.35% [54]. So one can obtain 12.99–21.65 μg Se, which verify that tea is an important source of Se.

The next-generation sequencing technologies are reliable tools to illuminate new genes and their involvement in biochemical pathways in non-model plants. Using RNA-seq, Se-induced genes have been identified in other species [55–56], but little has been known about genes involved in Se uptake, accumulation and tolerance in tea plant until recently. In this study, RNA-seq was employed to investigate Se genes based on expression changes in response to selenite, a predominant form of Se in the tea garden soil, and more than 1000 DEGs were identified in roots and leaves of tea plants. These DEGs, including some genes which had been reported previously, provided candidate genes for further investigating selenite uptake, transport and assimilation in tea plant and other plants. Furthermore, among the assembled unigenes, 78,806 were not annotated, which provided basic information on the discovery of new transcripts.

### Unigenes related to absorption, transport and metabolism during selenite treatment

**Fig 7:**
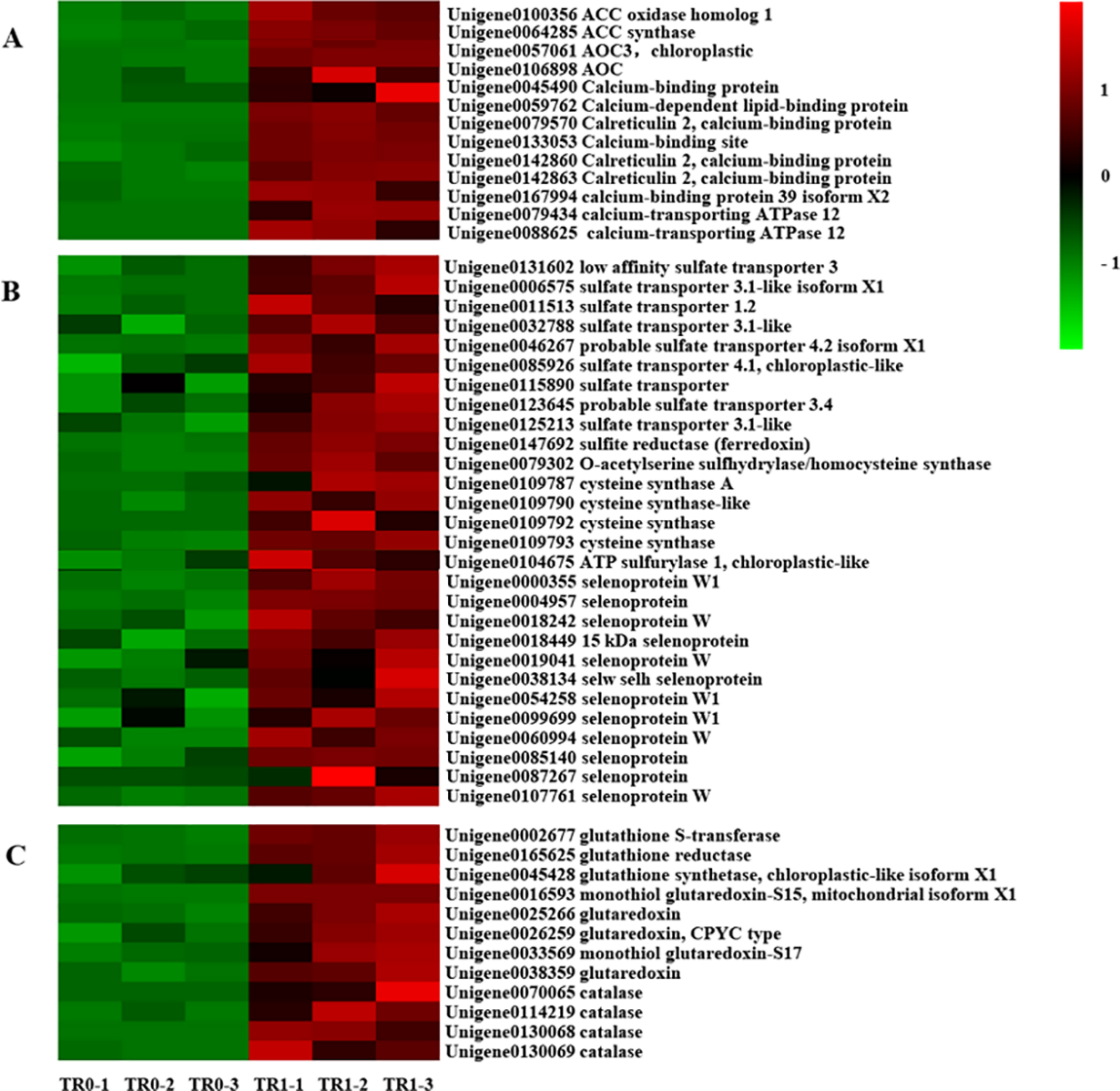
**Heat map diagram of expression patterns for DEGs related with signaling (A), sulfur and selenocompound metabolism (B), antioxidant control genges (C).** Colors show the log2(fold change value):the Redder the color,the higher the expression,the greener the color,the lower the expression.TR0-1,TR0-2,TR0-3:roots of control samples;TR1-1,TR1-2,TR1-3:roots of selenite treated samples.

Early studies implied that Se mainly as selenate and selenite is transported in plants via S and phosphate (P) transports, respectively. In our study, a comparison of the expression levels of genes encoding ion transporters was made between two treatments with and without selenite. Interestingly, genes encoding phosphate transporters were far more than those encoding sulfate transporters and expressed at significantly higher levels, though the absorption and translocation of selenite were not affected by sulphate [26]. There were 9 genes involved with sulfate transporters (Fig 7B). Besides, 18 phosphate transporter genes were memorably up-expressed in the roots, including mitochondrial phosphate carrier genes, inorganic phosphate transporter genes and phosphate transporter genes (Table 2). Among them, there were 3 up-regulated genes (Unigene0009567, Unigene0115349, Unigene0143441) encoding phosphate transporter PHO1-like protein, which were associated with ion transport between roots and shoots [57]. These results indicated P transporters and S transporters were responsible for the uptake and transport of selenite in tea plants, and P transporters played a more important role.

**Table 2:** The genes related with phosphate transporters in the roots of tea plants.

GeneID	Tissue	Description	Fold change	P value
Unigene0005774	roots	mitochondrial phosphate transporter	2.835	0.0073
Unigene0009567	roots	phosphate transporter PHO1 homolog 1	1.312	2.60E-05
Unigene0009898	roots	MC family transporter: phosphate	1.014	0.0028
Unigene0012455	roots	mitochondrial phosphate carrier 1, minor isoform	11.493	2.55E-12
Unigene0014471	roots	mitochondrial phosphate transporter	1.267	0.0057
Unigene0028463	roots	mitochondrial phosphate carrier 1	2.674	0.0152
Unigene0031729	roots	Mitochondrial phosphate carrier protein	1.567	5.55E-07
Unigene0058100	roots	mitochondrial phosphate transporter	11.710	4.52E-28
Unigene0066161	roots	phosphate transporter 1	1.879	4.41E-19
Unigene0067345	roots	mitochondrial phosphate transporter	3.815	1.20E-44
Unigene0083486	roots	mitochondrial phosphate carrier protein 2	11.397	6.88E-08
Unigene0083534	roots	high affinity inorganic phosphate transporter	2.664	4.35E-71
Unigene0096431	roots	Mitochondrial phosphate carrier protein	2.133	1.36E-26
Unigene0099537	roots	mitochondrial phosphate carrier protein 1	1.599	2.31E-07
Unigene0115349	roots	Phosphate transporter PHO1 -like protein	1.764	0.0099
Unigene0143441	roots	phosphate transporter PHO1-like protein	1.624	1.65E-24
Unigene0146768	roots	phosphate transport protein	2.641	0.0022
Unigene0146769	roots	phosphate transport protein	3.147	7.25E-05

Once absorbed into cells, Se can take advantage of S assimilation pathway and form into selenocystein and selenomethionine [58]. 34 different S-assimilation genes were significantly differentially expressed, including sulfate permease genes, sulfite reductase, ATP sulfurylase, and genes mediating cysteine and O-acetylserine sulfhydrylase/homocysteine synthesis genes in the roots, and 1 S-assimilation gene involved in cysteine synthesis had a higher expression in the leaves. ATP sulfurylase (*ATPS*) is the first and rate-limiting enzyme in the sulfate metabolic pathway. In our study, *ATPS1* was identified in the roots of tea plants (Fig 7B). There are four *ATPS* genes (*ATPS1-4*) in *Arabidopsis thaliana*, *ATPS2* is located in cytoplasm and plays a major role in the assimilation of selenate. *ATPS1*, *3*, *4* are found in plastid and provide *APS* for subsequent reactions but don’t participate in the reduction of Se [59]. Sulfite reductase is responsible for the reduction of selenite, and it has been identified to localize to plastids in *Arabidopsis* [60–61]. In leaves, Se accumulation might be related with the up-regulation of the genes encoding cysteine synthase, which was similar to the observations with other species [23, 62]. Moreover, 12 genes encoding selenoprotein were up-regulated in selenite treatment (Fig 7B), which was similar to the study by Freeman JL [62]. Selenocysteine was encoded by a UGA opal codon, which was generallya translational stop codon [63]. Selenoproteins that have selenocysteine residues in their catalytic site have been found in many organisms such as humans and bacteria but are rare in higher plants. Surprisingly, several selenoproteins were found in our study, which is worth our continued attention. In addition, 2 genes (Unigene0019110, Unigene0055824) encoding metallothioneins were up-regulated in the roots and 1 gene in the leaves (Unigene0019110). Previous studies have found that metallothioneins could affect metal tolerance and active oxygen scavenging [64].

### Unigenes related to antioxidant enzymes and antioxidant substances responding to selenite treatment

Another possible mechanism of Se tolerance and accumulation in tea plants might be its capacity to reduce or prevent Se-related oxidative stress. In the roots of tea plants grown with selenite treatment, 121 different genes were significantly expressed, encoding glutathione S-transferase (*GST*), glutathione synthetase, glutathione peroxidase, glutathione reductase, glutaredoxin and catalase. Meanwhile, 5 antioxidant-related genes showed significant expression in the leaves, encoding *GST*, catalase and defensin-like protein, which were considered to be Se-responsive genes in *Arabidopsis thaliana* and other Se-rich plants [62, 65]. It was worth mentioning that several *GST* genes were significantly expressed in both roots and leaves, and the highest abundance existed in roots rather than leaves. It was well known that *GST* was able to protect cells from oxidative damages because it could combine excess toxin with glutathion and form, transfer to and separate S-glutathione conjugates in the vacule [66]. Moreover, in roots, an up-regulated gene (Unigene0045428) encoding glutathione synthetase was deemed to be associated with Se tolerance and accumulation in tea plants. The reason was that *GSH* was related to selenite reduction and led to the absorption of selenite in roots [67]. Besides, 3 genes (Unigene0035324, Unigene0038126, Unigene0093881) encoding *GPX* were up-regulated, which might result in a high tolerance to selenite.

### Unigenes related to plant hormones in response to selenite treatment

Plant hormones are particularly important for nutrient homeostasis [68]. In our study, a certain number of genes in relation to plant hormones were found in roots, including 2 up-regulated genes (Unigene0100356, Unigene0064285) involved in ethylene biosynthesis and 2 up-regulated genes (Unigene0057061, Unigene0106898) involved in JA biosynthesis (Fig 7A). Previous studies have found that the levels of methyjasmonate (MeJA) and ethylene in young leaves were higher in *S*.*pinnata* (Se hyperaccumulator) than in *S*.*albescens* (secondary Se accumulator) with Se treatment [65]. In *Arabidopsis*, several genes related with the synthesis of jasmonate and ethylene were up-regulated in roots with Se treatment, which were proved to be associated with selenite resistance [68]. However, Zhou [31] reported that MeJA could effectively increase the absorption of selenate, but not selenite. In general, ethylene and jasmonate were thought to be “stress hormones”. Ethylene upregulated stress-related genes and JA upregulated both stress-related and S metabolism genes [69–70]. Generally, production of “stress hormones” is triggered by reactive oxygen species (ROS) [65, 71–72]. Under Se treatment, ROS was detected in both Se hyper-accumulator and non-accumulator plants [62, 65, 73]. An interesting thing was that ROS production induced by selenite was more in Col-0 (Se-resistant accession) than in Ws-2 (Se-sensitive accession) [65], which showed that ROS promoted the acquisition of Se resistance in non-accumulator plants. However, excessive ROS may suppress Se resistance. The generation of ROS might be caused by cytosolic calcium [58]. In our study, 9 genes related to calcium signaling, such as calcium-binding protein and calcium tranporters (Fig 7A), were identified in the roots, which was similar to Se-treated *A*.*thaliana* [74]. A possible mechanism is that the concentration changes of cytosolic calcium can improve NADPH oxidase activity, and trigger ROS generation [58]. However, selenite had an opposite effect on cytokinin and nitric oxide metabolism, and their overproduction led to selenite insensitivity [75]. Plant hormones, especially ethylene and jasmonate, could regulate a defensive network by up-regulating the expression levels of transcriptional factors. The genes encoding the ethylene-responsive factor (*EFR*) family (Unigene0060078, Unigene0106367, Unigene0149192) were identified in roots with selenite treatment. Moreover, two transcription factors belonging to the *MYB* transcription factor family (Unigene0088856, Unigene0094961) were up-regulated. Similarly, selenite treatment also induced the up-regulated expression of a heat shock transcription factor (Unigene0010336) and a bZIP reanscription factor (Unigene0039326) (Table 3). Furthermore, these hormones affected S uptake and assimilation [71], which might be helpful for plants to keep Se from replacing S in proteins [76].

**Table 3:** The genes related with transcription factor in the roots of tea plants.

GeneID	Tissue	Description	Fold change	P value
Unigene0010336	roots	Heat Shock transcription factor	4.442	6.40E-19
Unigene0039326	roots	bZIP transcription factor a	10.709	6.39E-09
Unigene0088856	roots	myb-related transcription factor	4.823	2.10E-20
Unigene0094961	roots	transcription factor MYB86	3.949	1.43E-12
Unigene0106367	roots	ethylene-responsive transcription factor 13-like	4.043	7.48E-18
Unigene0149192	roots	Ethylene-responsive transcription factor WIN1	4.674	8.46E-27
Unigene0060078	roots	AP2 domain-containing transcription factor family protein	4.994	7.43E-09

## Conclusion

As suggested by the RNA-seq analysis, selenite was mainly taken up by the phosphate transporters, most of which was stored in the roots of tea plants and then assimilated into organic forms such as selenocysteine through the sulfate assimilation pathway. The process might occur in both roots and leaves, mainly in roots. Selenite could induce substantial up-regulation of genes associated with oxidative stress, which suggested that antioxidant processes played an important role for Se tolerance and accumulation in tea plants. Moreover, hormones might play a signaling role. Our results provide valuable information on the molecular regulation of selenite in tea plant.

## Supporting information

S1 Fig Changes of tea plants in response to selenite treatment. (A) Regular of Se enrichment characteristic in the roots of tea plants (Different letters represent significant difference at 0.01 level). (B) Morphological appearance of tea plant leaves treated with the gradient selenite of 0, 0.015, 0.025, 0.05, 0.1, 0.2, 0.4 mmol/L.
(TIF)

S2 Fig Lenth distribution of *Camellia sinensis* unigenes. (TIFF)

S3 Fig Number of significantly DEGs after selenite treatment in roots (TR0-VS-TR1) and leaves (TL0-VS-TL1). The red bars represent up-regulated genes, and the blue bars represent down-regulated genes (FDR<0.05, |log2ratio|>1).
(TIFF)

S4 Fig Function classifications of GO terms of tea plant leaves transcripts. The y-axis indicates the number of genes in a subcategory, and the x-axis indicates the different subcategories.
(TIFF)

S1 Table Primers used for qRT-PCR. (DOCX)

S2 Table The results of raw reads filtering. TL0-1,TL0-2,TL0-3:leaves of control samples;TL1-1,TL1-2,TL1-3:leaves of selenite treated samples;TR0-1,TR0-2,TR0-3:roots of control samples;TR1-1,TR1-2,TR1-3:roots of selenite treated samples.
(DOCX)

S3 Table The results of *de novo* assembly. (DOCX)

S4 Table The most affected pathways and genes in the roots and leaves of tea plants. (DOC)
