# Supplementary figures and images for: Transcriptome analysis of differentially expressed genes involved in selenium accumulation in tea plant (Camellia sinensis)
Source: PLoS One. 2018 Jun 1;13(6):e0197506. doi: 10.1371/journal.pone.0197506 (PMC5983420; doi:10.1371/journal.pone.0197506)

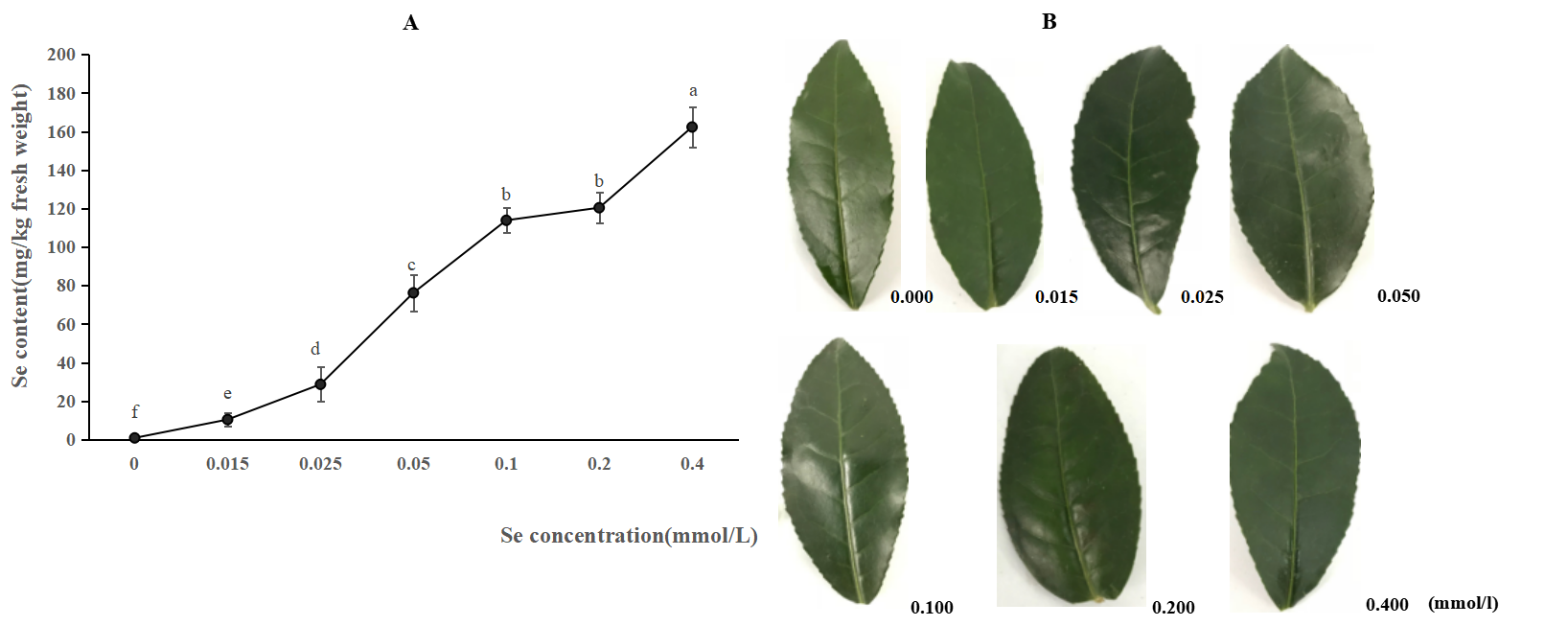

Supplement: S1 Fig — (A) Regular of Se enrichment characteristic in the roots of tea plants (Different letters represent significant difference at 0.01 level). (B) Morphological appearance of tea plant leaves treated with the gradient selenite of 0, 0.015, 0.025, 0.05, 0.1, 0.2, 0.4 mmol/L. (TIF) [file pone.0197506.s001.tif]

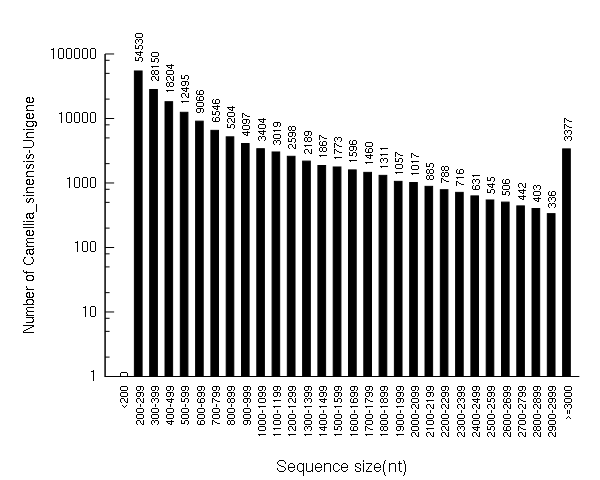

Supplement: S2 Fig — (TIFF) [file pone.0197506.s002.tiff]

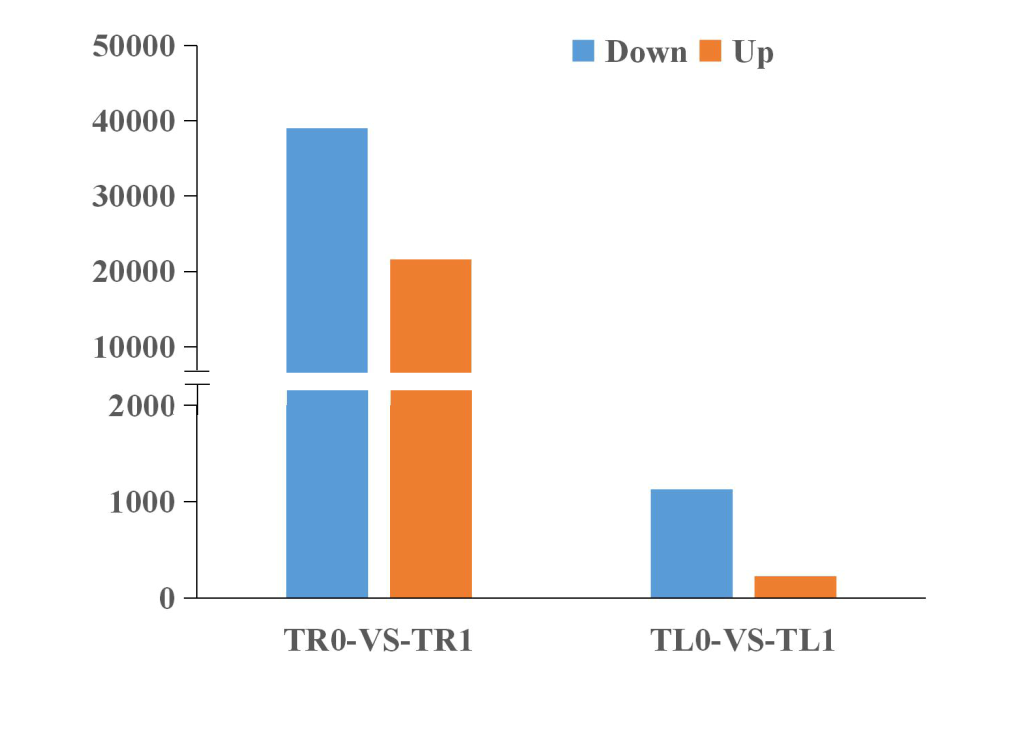

Supplement: S3 Fig — The red bars represent up-regulated genes, and the blue bars represent down-regulated genes (FDR<0.05, |log2ratio|>1). (TIFF) [file pone.0197506.s003.tiff]

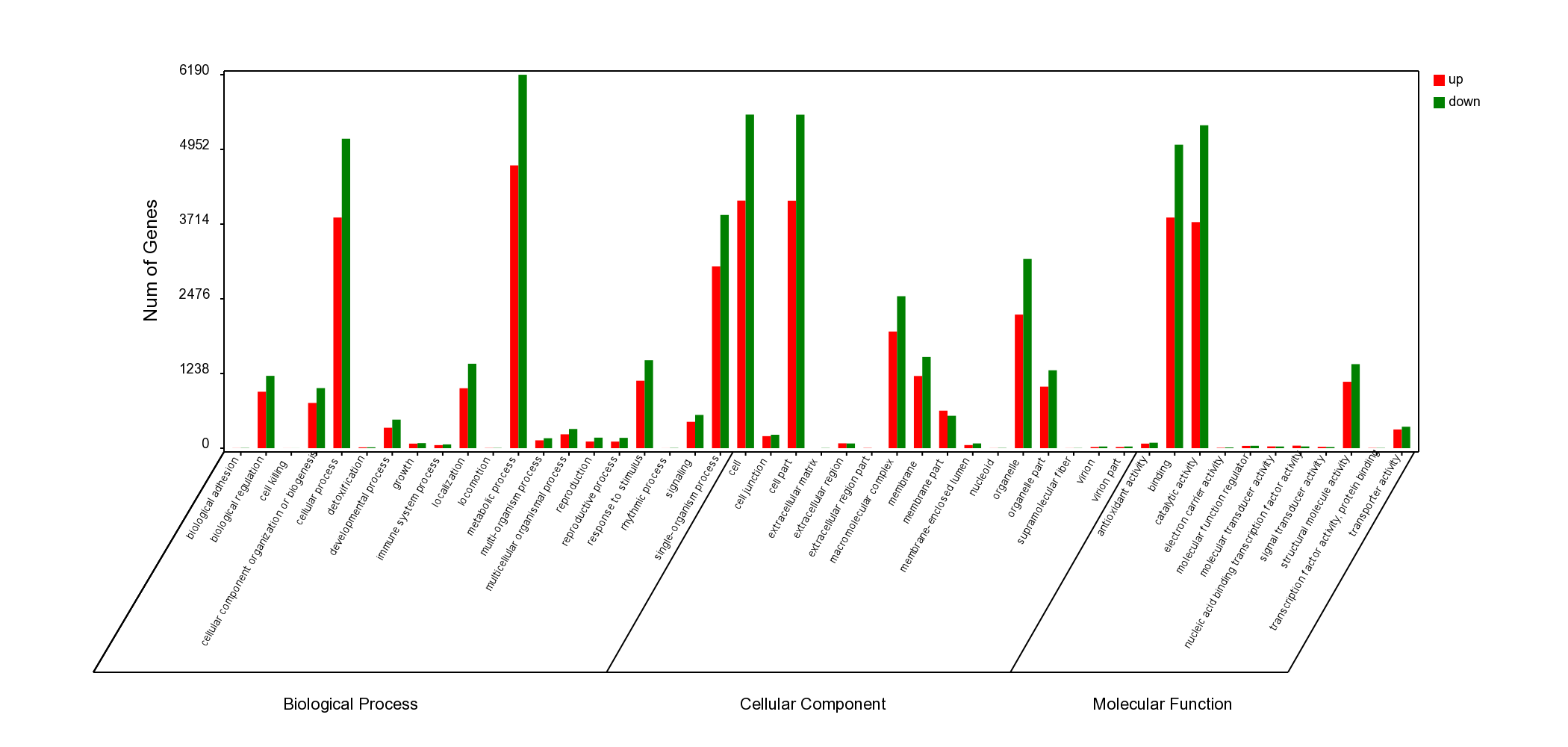

Supplement: S4 Fig — The y-axis indicates the number of genes in a subcategory, and the x-axis indicates the different subcategories. (TIFF) [file pone.0197506.s004.tiff]
