# Supplementary material for: Transcriptome analysis of differentially expressed genes involved in selenium accumulation in tea plant (Camellia sinensis)
Source: PLoS One. 2018 Jun 1;13(6):e0197506. doi: 10.1371/journal.pone.0197506 (PMC5983420; doi:10.1371/journal.pone.0197506)
Supplement: S1 Table — (DOCX) [file pone.0197506.s005.docx]

**S1 Table. Primers used for qRT-PCR.**

| Unigenes ID | annotation | Primer (F) | Primer (R) |
| --- | --- | --- | --- |
|  |  |  |  |
| Unigene0014190 | ATP sulfurylase | CAGTCTGGAGATGGTGG | CCTTCTTCAGGTCGTCG |
| Unigene0165507 | 1-aminocyclopropane-1-carboxylate synthase, partial | GGATGTGGTGGATGTCT | TGAGCAGCACGATTATT |
| Unigene0079302 | O-acetylserine sulfhydrylase/homocysteine synthase | CATCGGTGGTGTCATCAT | ACATCCCAGAACTTCAGT |
| Unigene0109787 | cysteine synthase A | AGAGCCCTGTGCTTTCG | CGGTGATGACCTCGTCG |
| Unigene0109792 | PREDICTED: cysteine synthase | GAGTTCATGGAGCCCTG | TGTGCGTATCCTTGGTG |
| Unigene0165625 | glutathione reductase | GCATCAATGTGTCTGGC | CGACTTTGTTTGGTAGG |
| Unigene0038596 | microsomal Glutathione S-transferase | CACTACCCGCTGCCTTCT | AGTTTCCACGCATTCGCT |
| Unigene0026184 | catalase/peroxidase | CGCCTCATGGGCTTCTCG | TGGACAGCAGGGACGGGT |
| Unigene0070065 | catalase | GGGATTTGAAGGAGGAT | CCAATAAAGACAGAGTA |
| Unigene0035741 | monodehydroascorbate reductase, seedling isozyme | TAGTTGTTGTGGGGGCG | CGGTTTCAAGTTCGGTG |
| Unigene0012455 | mitochondrial phosphate carrier 1, minor isoform | TGTGCCTCCGCCTCAGCC | CCAAGCATCCAGTTCTCG |
| Unigene0058100 | mitochondrial phosphate transporter | GGGCTCCTACTTTTATT | TTATTCGCATTTTCTTC |
| Unigene0083486 | PREDICTED: LOW QUALITY PROTEIN: mitochondrial phosphate carrier protein 2, mitochondrial-like | TTGGTCCTCGTGTGTTCA | GCAGTGACAGCCTTGACA |
| Unigene0039326 | bZIP transcription factor a | TAGGAGATTGGTAAAGAA | TATCAGAGGTCAGAGTTG |
| Unigene0060078 | AP2 domain-containing transcription factor family protein | TGTCGCCGAGTCAGATT | ATAGAGCCCCAGCCAGT |
| β-actin |  | GCCATCTTTGATTGGAATGG | GGTGCCACAACCTTGATCTT |
| GAPDH |  | TTGGCATCGTTGAGGGTCT | CAGTGGGAACACGGAAAGC |
