# Supplementary material for: Transcriptome analysis of differentially expressed genes involved in selenium accumulation in tea plant (Camellia sinensis)
Source: PLoS One. 2018 Jun 1;13(6):e0197506. doi: 10.1371/journal.pone.0197506 (PMC5983420; doi:10.1371/journal.pone.0197506)
Supplement: S2 Table — TL0-1,TL0-2,TL0-3:leaves of control samples;TL1-1,TL1-2,TL1-3:leaves of selenite treated samples;TR0-1,TR0-2,TR0-3:roots of control samples;TR1-1,TR1-2,TR1-3:roots of selenite treated samples. (DOCX) [file pone.0197506.s006.docx]

**S2 Table. The results of raw reads filtering**

| sequences | Total raw reads(million) | Total clean reads  (million) | Q20 % | GC(%) |
| --- | --- | --- | --- | --- |
| TL0-1 | 34.08 | 33.00(96.84%) | 96.89 | 44.23 |
| TL0-2 | 31.1 | 30.04(96.59%) | 96.86 | 44.41 |
| TL0-3 | 32.9 | 31.91(97%) | 96.97 | 43.77 |
| TL1-1 | 33.01 | 31.80(96.35%) | 96.73 | 43.73 |
| TL1-2 | 31.97 | 31.03(97.04%) | 96.86 | 44.35 |
| TL1-3 | 38 | 36.85(96.97%) | 96.89 | 43.94 |
| TR0-1 | 33.81 | 32.72(96.78%) | 96.77 | 45.6 |
| TR0-2 | 40.95 | 39.68(96.9%) | 96.78 | 45.75 |
| TR0-3 | 31.54 | 30.40(96.39%) | 96.61 | 45.98 |
| TR1-1 | 30.5 | 29.47(96.63%) | 96.72 | 44.68 |
| TR1-2 | 36.3 | 35.13(96.78%) | 96.84 | 44.66 |
| TR1-3 | 37.06 | 35.95(97%) | 96.91 | 44.75 |

TL0-1,TL0-2,TL0-3:leaves of control samples;TL1-1,TL1-2,TL1-3:leaves of selenite treated samples;TR0-1,TR0-2,TR0-3:roots of control samples;TR1-1,TR1-2,TR1-3:roots of selenite treated samples
