# Supplementary material for: Transcriptome analysis of differentially expressed genes involved in selenium accumulation in tea plant (Camellia sinensis)
Source: PLoS One. 2018 Jun 1;13(6):e0197506. doi: 10.1371/journal.pone.0197506 (PMC5983420; doi:10.1371/journal.pone.0197506)
Supplement: S3 Table — (DOCX) [file pone.0197506.s007.docx]

**S3 Table. The results of *de novo* assembly**

| Genes Num | GC percentage | N50 | Max length | Min length | Average length |
| --- | --- | --- | --- | --- | --- |
| 168212 | 43.4846 | 997 | 17995 | 201 | 677 |
