# Supplementary material for: Transcriptome analysis of differentially expressed genes involved in selenium accumulation in tea plant (Camellia sinensis)
Source: PLoS One. 2018 Jun 1;13(6):e0197506. doi: 10.1371/journal.pone.0197506 (PMC5983420; doi:10.1371/journal.pone.0197506)
Supplement: S4 Table — (DOC) [file pone.0197506.s008.doc]

**S4 Table. The most affected pathways and genes in the roots and leaves of tea plant.**

| Accession | Tissue | Description | Fold change | P value |
| --- | --- | --- | --- | --- |
| **Ethylene synthesis** | | | | |
| Unigene0100356 | roots | PREDICTED: LOW QUALITY PROTEIN: 1-aminocyclopropane-1-carboxylate oxidase homolog 1 | 1.680 | 3.13E-12 |
| Unigene0064285 | roots | 1-aminocyclopropane-1-carboxylate synthase | 1.919 | 1.28E-28 |
| **JA synthesis** | | | | |
| Unigene0057061 | roots | PREDICTED: allene oxide cyclase 3, chloroplastic | 1.315 | 1.80E-32 |
| Unigene0106898 | roots | allene oxide cyclase | 1.116 | 4.73E-44 |
| **Sulfur metabolism** | | | | |
| Unigene0131602 | roots | PREDICTED: low affinity sulfate transporter 3 | 3.065 | 2.41E-09 |
| Unigene0006575 | roots | PREDICTED: sulfate transporter 3.1-like isoform X1 | 2.595 | 3.36E-20 |
| Unigene0011513 | roots | sulfate transporter 1.2 | 3.121 | 8.71E-13 |
| Unigene0032788 | roots | PREDICTED: sulfate transporter 3.1-like | 1.663 | 0.0042 |
| Unigene0046267 | roots | PREDICTED: probable sulfate transporter 4.2 isoform X1 | 3.409 | 3.50E-21 |
| Unigene0085926 | roots | PREDICTED: sulfate transporter 4.1, chloroplastic-like | 2.058 | 0.0193 |
| Unigene0115890 | roots | sulfate transporter | 1.764 | 0.0024 |
| Unigene0123645 | roots | PREDICTED: probable sulfate transporter 3.4 | 2.890 | 8.38E-06 |
| Unigene0125213 | roots | PREDICTED: sulfate transporter 3.1-like | 2.033 | 4.24E-09 |
| Unigene0099386 | roots | Sulfite reductase [ferredoxin] SIR | 2.524 | 9.62E-05 |
| Unigene0099388 | roots | sulfite reductase | 1.825 | 0.0005 |
| Unigene0132322 | roots | sulfite reductase (NADPH) flavoprotein alpha-component | 2.222 | 0.0010 |
| Unigene0147691 | roots | sulfite reductase | 4.317 | 5.05E-21 |
| Unigene0147692 | roots | sulfite reductase (ferredoxin) | 4.542 | 4.38E-78 |
| Unigene0079302 | roots | O-acetylserine sulfhydrylase/homocysteine synthase | 5.130 | 1.98E-13 |
| Unigene0109787 | roots | cysteine synthase A | 5.108 | 7.88E-07 |
| Unigene0109790 | roots | PREDICTED: cysteine synthase-like | 3.540 | 2.88E-07 |
| Unigene0109792 | roots | PREDICTED: cysteine synthase | 9.938 | 0.0002 |
| Unigene0109793 | roots | cysteine synthase | 2.745 | 1.28E-21 |
| Unigene0014190 | roots | ATP sulfurylase | 5.578 | 7.5E-10 |
| Unigene0104674 | roots | ATP sulfurylase | 2.570 | 4.66E-05 |
| Unigene0104675 | roots | PREDICTED: ATP sulfurylase 1, chloroplastic-like | 2.001 | 0.0058 |
| Unigene0104676 | roots | ATP sulfurylase | 2.067 | 0.0035 |
| **Antioxidant and redox control genges** | | | | |
| Unigene0001927 | roots | PREDICTED: probable glutathione S-transferase parA | 1.760 | 3.38E-17 |
| Unigene0002677 | roots | glutathione S-transferase | 2.822 | 8.08E-18 |
| Unigene0003458 | roots | PREDICTED: glutathione S-transferase omega-like 2 | 1.888 | 8.67E-18 |
| Unigene0004158 | roots | PREDICTED: glutathione S-transferase F9 | 1.708 | 2.75E-15 |
| Unigene0004261 | roots | glutathione S-transferase | 2.459 | 6.37E-32 |
| Unigene0165625 | roots | glutathione reductase | 5.687 | 7.68E-72 |
| Unigene0045428 | roots | PREDICTED: glutathione synthetase, chloroplastic-like isoform X1 | 1.584 | 0.0072 |
| Unigene0010141 | roots | glutaredoxin, CPYC type | 1.575 | 3.52E-13 |
| Unigene0016593 | roots | PREDICTED: monothiol glutaredoxin-S15, mitochondrial isoform X1 | 2.602 | 8.96E-198 |
| Unigene0025266 | roots | PREDICTED: glutaredoxin | 3.453 | 1.49E-20 |
| Unigene0026259 | roots | glutaredoxin, CPYC type | 2.796 | 0.0008 |
| Unigene0033569 | roots | PREDICTED: monothiol glutaredoxin-S17 | 4.050 | 5.47E-06 |
| Unigene0038359 | roots | PREDICTED: glutaredoxin | 3.828 | 4.76E-12 |
| Unigene0003446 | roots | catalase | 1.341 | 0.0051 |
| Unigene0007325 | roots | catalase | 1.519 | 0.0022 |
| Unigene0007959 | roots | catalase | 2.738 | 3.07E-06 |
| Unigene0015335 | roots | catalase | 3.613 | 2.01E-39 |
| Unigene0026181 | roots | Catalase decomposes hydrogen peroxide to molecular oxygen and water | 2.336 | 0.0001 |
| Unigene0026182 | roots | catalase/peroxidase | 3.114 | 0.0010 |
| Unigene0026183 | roots | catalase | 3.881 | 2.53E-07 |
| Unigene0034053 | roots | catalase | 1.269 | 0.0061 |
| Unigene0047791 | roots | Catalase | 1.837 | 0.0030 |
| Unigene0070065 | roots | catalase | 10.957 | 1.16E-06 |
| Unigene0074977 | roots | catalase | 3.338 | 0.0044 |
| Unigene0078864 | roots | Catalase | 2.693 | 8.42E-14 |
| Unigene0078865 | roots | catalase | 3.476 | 1.31E-08 |
| Unigene0085730 | roots | catalase | 3.315 | 0.0205 |
| Unigene0089043 | roots | catalase | 1.231 | 5.89E-05 |
| Unigene0114219 | roots | catalase | 4.347 | 4.4E-08 |
| Unigene0130068 | roots | catalase | 11.962 | 1.86E-32 |
| Unigene0130069 | roots | catalase | 6.458 | 3.42E-25 |
| Unigene0035324 | roots | glutathione peroxidase | 1.928 | 3.67E-06 |
| Unigene0038126 | roots | PREDICTED: probable glutathione peroxidase 4 | 1.935 | 3.23E-05 |
| Unigene0093881 | roots | glutathione peroxidase | 9.600 | 0.0010 |
| Unigene0047909 | leaves | Catalase | 2.179 | 0.0009 |
| Unigene0019110 | leaves | PREDICTED: metallothionein-like protein 1 | 1.164 | 2.47E-37 |
| **Amino acid metabolism** | | | | |
| Unigene0006957 | roots | amino acid transporter, AAAP family | 4.515 | 2.86E-23 |
| Unigene0007069 | roots | PREDICTED: proton-coupled amino acid transporter 3 | 5.987 | 1.11E-48 |
| Unigene0008829 | roots | amino acid transporter, AAAP family | 3.795 | 6.15E-43 |
| Unigene0010037 | roots | PREDICTED: cationic amino acid transporter 5-like | 3.992 | 1.12E-12 |
| Unigene0031441 | roots | PREDICTED: vacuolar amino acid transporter 1-like | 3.792 | 3.23E-60 |
| Unigene0032716 | roots | cationic amino acid transporter 2, vacuolar protein | 3.481 | 5.58E-11 |
| Unigene0074797 | roots | cationic amino acid transporter-like protein | 11.242 | 1.76E-09 |
| Unigene0080491 | roots | amino acid transporter | 1.571 | 1.19E-06 |
| Unigene0080492 | roots | amino acid transporter | 1.499 | 0.0014 |
| Unigene0082126 | roots | PREDICTED: sodium-coupled neutral amino acid transporter 2-like | 1.239 | 1.33E-09 |
| Unigene0102169 | roots | PREDICTED: vacuolar amino acid transporter 1-like | 1.014 | 0.0206 |
| Unigene0127065 | roots | amino acid transporter | 4.889 | 5.38E-68 |
| Unigene0127069 | roots | amino acid transporter | 11.309 | 2.61E-12 |
| Unigene0142557 | roots | PREDICTED: vacuolar amino acid transporter 1 | 4.431 | 2.00E-207 |
| Unigene0008502 | roots | serine hydroxymethyltransferase | 1.937 | 3.73E-07 |
| Unigene0022063 | roots | serine hydroxymethyltransferase | 9.925 | 0.0007 |
| Unigene0023398 | roots | PREDICTED: serine hydroxymethyltransferase 4-like, partial | 2.478 | 0.0128 |
| Unigene0050614 | roots | Pyridoxal-phosphate-dependent serine hydroxymethyltransferase | 2.944 | 1.40E-05 |
| Unigene0073204 | roots | serine hydroxymethyltransferase | 1.978 | 0.0057 |
| Unigene0129260 | roots | glycine/serine hydroxymethyltransferase | 1.217 | 7.05E-09 |
| Unigene0130208 | roots | serine hydroxymethyltransferase | 4.352 | 2.56E-62 |
| Unigene0082464 | roots | branched-chain amino acid aminotransferase | 5.209 | 4.27E-08 |
| **selenoproteins** | | | | |
| Unigene0000355 | roots | selenoprotein W1 | 2.351 | 8.92E-09 |
| Unigene0004957 | roots | selenoprotein | 4.463 | 1.42E-18 |
| Unigene0018242 | roots | selenoprotein W | 2.433 | 1.26E-08 |
| Unigene0018449 | roots | 15 kDa selenoprotein | 1.690 | 6.29E-05 |
| Unigene0019041 | roots | selenoprotein W | 1.102 | 0.0006 |
| Unigene0038134 | roots | selw selh selenoprotein | 3.732 | 9.44E-07 |
| Unigene0054258 | roots | selenoprotein W1 | 1.715 | 0.0038 |
| Unigene0099699 | roots | selenoprotein W1 | 1.777 | 0.0005 |
| Unigene0060994 | roots | selenoprotein W | 3.846 | 9.91E-10 |
| Unigene0085140 | roots | selenoprotein | 2.681 | 9.42E-08 |
| Unigene0087267 | roots | selenoprotein | 2.696 | 0.0118 |
| Unigene0107761 | roots | selenoprotein W | 2.544 | 3.56E-18 |
| **Calcium signal** | | | | |
| Unigene0045490 | roots | PREDICTED: probable calcium-binding protein | 3.988 | 2.82E-13 |
| Unigene0059762 | roots | Calcium-dependent lipid-binding protein | 3.649 | 8.65E-07 |
| Unigene0079570 | roots | Calreticulin 2, calcium-binding protein | 3.947 | 2.85E-152 |
| Unigene0133053 | roots | Calcium-binding site | 3.302 | 1.49E-107 |
| Unigene0142860 | roots | Calreticulin 2, calcium-binding protein | 4.042 | 1.29E-21 |
| Unigene0142863 | roots | Calreticulin 2, calcium-binding protein | 3.199 | 2.29E-41 |
| Unigene0167994 | roots | PREDICTED: calcium-binding protein 39 isoform X2 | 5.068 | 4.71E-12 |
| Unigene0079434 | roots | PREDICTED: calcium-transporting ATPase 12, plasma membrane-type-like | 9.305 | 0.0043 |
| Unigene0088625 | roots | PREDICTED: calcium-transporting ATPase 12, plasma membrane-type | 11.494 | 8.87E-12 |
